# Quantitative Analysis of the Processes and Signaling Events Involved in Early HIV-1 Infection of T Cells

**DOI:** 10.1371/journal.pone.0103845

**Published:** 2014-08-08

**Authors:** Guido Santos, Agustín Valenzuela-Fernández, Néstor V. Torres

**Affiliations:** 1 Grupo de Biología de Sistemas y Modelización Matemática, Departamento de Bioquímica, Microbiología, Biología Celular y Genética, Facultad de Biología, Universidad de La Laguna, San Cristóbal de La Laguna, Tenerife, España; 2 Laboratorio de Inmunología Celular y Viral, Departamento de Medicina Física y Farmacología, Facultad de Medicina, Universidad de La Laguna, San Cristóbal de La Laguna, Tenerife, España; 3 Instituto de Tecnología Biomédica, Universidad de La Laguna, San Cristóbal de La Laguna, Tenerife, Spain; George Mason University, United States of America

## Abstract

Lymphocyte invasion by HIV-1 is a complex, highly regulated process involving many different types of molecules that is prompted by the virus's association with viral receptors located at the cell-surface membrane that culminates in the formation of a fusion pore through which the virus enters the cell. A great deal of work has been done to identify the key actors in the process and determine the regulatory interactions; however, there have been no reports to date of attempts being made to fully understand the system dynamics through a systemic, quantitative modeling approach. In this paper, we introduce a dynamic mathematical model that integrates the available information on the molecular events involved in lymphocyte invasion. Our model shows that moesin activation is induced by virus signaling, while filamin-A is mobilized by the receptor capping. Actin disaggregation from the cap is facilitated by cofilin. Cofilin is inactivated by HIV-1 signaling in activated lymphocytes, while in resting lymphocytes another signal is required to activate cofilin in the later stages in order to accelerate the decay of the aggregated actin as a restriction factor for the viral entry. Furthermore, stopping the activation signaling of moesin is sufficient to liberate the actin filaments from the cap. The model also shows the positive effect of gelsolin on actin capping by means of the nucleation effect. These findings allow us to propose novel approaches in the search for new therapeutic strategies. In particular, gelsolin inhibition is seen as a promising target for preventing HIV-1 entry into lymphocytes, due to its role in facilitating the capping needed for the invasion. Also it is shown that HIV-1 should overcome the cortical actin barrier during early infection and predicts the different susceptibility of CD4+ T cells to be infected in terms of actin cytoskeleton dynamics driven by associated cellular factors.

## Introduction

The invasion and infection of CD4+ T lymphocytes by human immunodeficiency virus type 1 (HIV-1) is a complex process involving many cellular events that have been the subject of many studies [1]. The accumulated evidence indicates that the actin mobilization that occurs before the formation of the fusion pore plays a central role in this process. In fact, the actin cytoskeleton is deeply involved in the capping of cell-surface receptors for viral infection, which facilitates the interaction with the viral envelope (Env) complex and the subsequent fusion pore formation. However, this is not the only cellular component of importance in the viral infection process. Another main character in this plot is the HIV-1 Env-gp120 viral-surface protein. This element, located in the virus's outer coat, docks with high affinity at the lymphocyte's surface CD4 receptor. As a consequence of this interaction, HIV-1 Env-gp120 changes its conformation, exposing other regions of the viral protein responsible for its binding to a second co-receptor, either CCR5 or CXCR4. These bindings trigger a signaling pathway inside the lymphocyte that culminates with the formation of an actin cap in a pole of the cell (hereinafter ‘cap’), driving CD4 and co-receptor co-localization and direct interaction, in an actin-dependent manner. These HIV-1 Env-gp120/CD4-mediated actin and receptor reorganization and capping events have been shown to correlate with the infectivity of the virus [2]. This fact will be a central issue the present study, since we will choose a cap indicator as a measure of HIV-1 infectivity.

Another observed fact is that activated CD4+ T lymphocytes, due to its active cell cycling, are continuously remodeling their actin cytoskeleton. There is ample evidence that the inhibition of the signal transduction or the removal of the intracellular signaling domain of CXCR4/CCR5, did not affect HIV infection [3]–[6]. However, in resting CD4+ T lymphocytes such inhibition diminishes HIV infection [7]. In the same vein it has been shown that resting T cells are more sensitive to actin inhibitors than transformed T cells [8]. All these evidences point out to the fact that while the viral requirement for actin dynamics are universal, the HIV-mediated signaling pathways to the actin activity are cell-line dependent. These facts have been taken into account in this modelling exercise.

The actin mobilization required for cap formation is in turn influenced by other elements. This is the case of moesin, for example, an HIV-1-activated protein that acts as a reversible link between the lymphocyte membrane and the actin filaments [9]. HIV-1-triggered moesin activation promotes the reorganization of cortical F-actin and its subsequent anchoring to the membrane at HIV-1-cell contact points [10]; through this interaction, it facilitates the receptor/co-receptor direct interaction and co-localization. Furthermore, moesin also promotes the polymerization of actin filaments as a nucleation factor [10]–[12]. Moreover, experimental results show that increasing the total moesin available at the lymphocyte enhances HIV-1 infectivity, while a decrease in the activity of moesin negatively affects the invasion process [10]. During fusion pore formation, moesin has to be deactivated to allow F-actin depolymerization and viral entry [13].

Other key players in these processes are gelsolin, filamin-A and cofilin. Gelsolin is an actin-binding protein with a severing activity on actin filaments, which thus also has an effect on actin mobilization. It is assumed that this severing activity is what underlies the protein's observed influence on virus infectivity, by driving HIV-1-mediated cortical actin reorganization [14]. Gelsolin acts as a basal restrictive barrier for HIV-1 infection by severing actin to control the appropriate amount of cortical actin to be reorganized together with CD4-CXCR4/CCR5 redistribution to one pole of the cell. Both events are required for limiting early viral infection [14]. In the case of filamin-A, this protein participates in the invasion by linking membrane receptors to the actin cytoskeleton [15]. It has been shown too, that changes in filamin-A activity affects the invasion process of HIV-1 [15].

Finally, the last element to be considered is the actin-severing factor cofilin. This protein is regulated by virus signaling through the CXCR4 co-receptor and LIMK activation that leads to cofilin phosphorylation and inactivation [7], [16], thus assuring an intact actin cortex before fusion pore formation. However, the mechanism involved in the activation of cofilin, just at the instant where the fusion pore is formed to allow cortical actin destruction and viral capsid entry, is not well understood. It has been observed that increasing the activity of cofilin enhances the infectivity of HIV-1 on resting lymphocytes, but that this does not have any effect on active lymphocytes [7]. In order to explain these observations, it has been hypothesized that cofilin facilitates cortical actin remodeling after fusion pore formation in resting lymphocytes only; this effect is caused by the impairment of the viral restriction factor at the static cortical actin in resting cells at later stages of the invasion [7], [17].

Furthermore, the complexity of this scenario is growing, as recently other actin-associated factors appear to alter early HIV-1 infection. Hence, RNA silencing of debrin decreases F-actin polymerization allowing HIV-1 infection [18], while syntenin-1 depolymerises F-actin in a post-fusion step [19]. Although the HIV-1 Env-mediated signaling that activates LIMK-cofilin appears to be more clear after the involvement of PAK1/2 and the role of LIMK in viral-induced actin capping, the factors that lies upstream the RhoA/Rac1-PAK1/2-LIMK-cofilin and syntenin-1 pathways remain poorly understood [19]–[23]. Similarly, the identity of the kinase that phosphorylates moesin in the ERM-F-actin/receptor complex is unknown [10]. The cap itself, together with the processes described above that lead to its formation, emerges in many studies [1], [2], [7], [10], [14], [15], [24] as one of the main system responses prompted by the HIV-1 signaling.

From this observation it can naturally be derived that any insight into this fairly complex dynamic phenomenon is of foremost interest. Consequently, a great deal of information has been accumulated on the factors influencing the cap formation [7], [10], [14], [15]. The approach followed when gathering the bulk of this information has been determined by the need to isolate the influence of each element considered relevant from that of the others participating in the process. In our view, these attempts to understand cap formation can be complemented by taking an integrated approach, where the activities of most of the key factors already described are simultaneously considered in a quantitative and dynamic framework. This integrated approach is not new in the field, since it has been used to unravel different aspects of HIV-1 infection [25]–[28]. However, to our knowledge, the present work is the first integrated exercise on the invasion of lymphocytes by HIV-1 during the first stages of the viral cycle.

Following this line of reasoning, the aim of this work is to integrate all available information on the molecules, mechanisms and regulatory features involved in the early lymphocyte invasion process into a dynamic mathematical model. By means of this approach, we aim to achieve a better understanding of the dynamics of the process and the role played by the various molecular components. The model is based on a plethora of experimental observations already made on the functional role of a number of cytoskeleton elements (receptors, enzymes, proteins, etc.) that participate in cytoskeleton reorganization and plasma membrane dynamics. It is this systemic approach that will allow us to model and evaluate the dynamics of the plasma membrane, as well as the role and relative importance of the different cortical structures and signal transduction through the CD4 receptor and CXCR4 or CCR5 co-receptors, which are the viral receptors involved in the generation of the membrane fluidity to promote fusion pore formation, entry and infection.

## Results and Discussion

The signaling structure involved in the actin mobilization observed throughout the first stages of lymphocyte invasion by HIV-1 has recently been elucidated in great detail (see Liu et al. 2009, [1]). Key actors in these series of events are the CD4 and CXCR4 (or CCR5) membrane receptors, filamin-A and the ERM protein moesin, actin and the severing factor cofilin, as well as gelsolin, another actin-severing factor. In order to unravel the role of each one of these molecules in the process, they have been studied separately [7], [10], [14], [15]. As a result, we have a considerable body of information offering a great deal of insight into the series of coordinated events involved in lymphocyte invasion by HIV-1.

**Figure 1:**
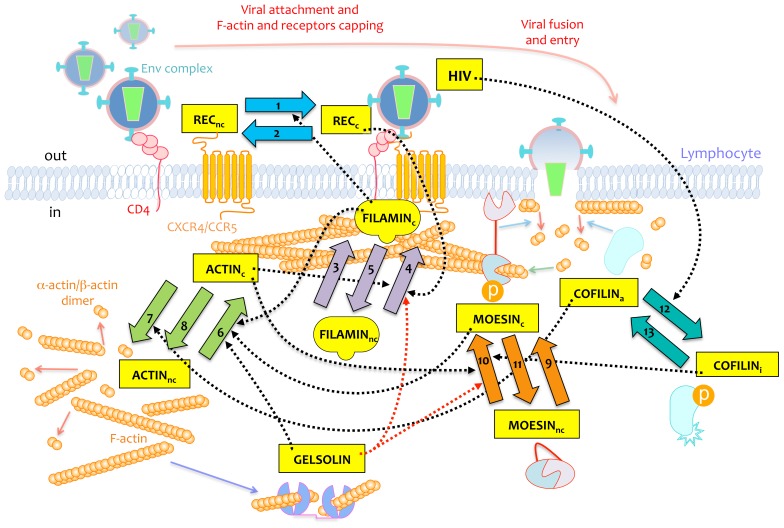
Representation of the molecular events simulated in the mathematical model. Molecules included in the model as variables are the following: HIV, REC (CD4 and CXCR4 or CCR5 receptors for HIV-1 infection on lymphocyte cell-surface), FILAMIN, MOESIN (phosphorylated and active; dephosphorylated and non-active), COFILIN (a, active; i, inactive), and ACTIN. Molecules recruited at the HIV-1-triggered capping regions are indicated by the c subscript, while non-capped molecules outside this region are indicated by the nc subscript. As it is assumed that gelsolin remains constant during the whole process, it is not incorporated as a variable in the model. Numbered arrows (from 1 to 13) are the processes included in the model, and dashed arrows are the interactions from the molecules to the processes (black are positive, red are negative). Gelsolin acts by remodeling the amount and size of actin filaments, so the total amount of actin and its reorganization is reduced by higher expression of gelsolin (negative influence of GELSOLIN on processes 4 and 10, see Material and Methods for details); furthermore, appropriate levels of gelsolin facilitate, through the orchestrated severing and remodeling of actin filaments, the capping of actin filaments at viral entry regions (positive effect of GELSOLIN on process 6). Continuous arrows serve as an additional explanation of molecular events taking place during the invasion. Thus, red arrows represent depolymerization of actin filaments, blue arrows represent components which assist the depolymerization of actin filaments (e.g., active cofilin and inactivation of moesin in fusion pore formation), the green arrow indicates actin monomer incorporation to the growing actin filaments, and the purple arrow represents the actin severing and remodeling by gelsolin, thereby controlling the size of actin filaments and the amount of filaments reorganized to the viral entry regions on the plasma membrane of target cells.

However, the existing descriptions and interpretations are in many cases “element biased”, since there is currently no integrated picture of the process where all the system components are simultaneously considered in a dynamic and quantitative way. In this work, we have tried to fill this gap by proposing a mathematical model where a great deal of the available information about the elements and the interactions among them is organized and integrated in a dynamic fashion (Figure 1).

The model thus obtained has been shown to be a robust and reliable representation of the system under consideration (see Material and Methods). Based on this model and on its subsequent analysis, we have been able to quantify the relative importance of each component for the system.

### Relevant processes

**Figure 2:**
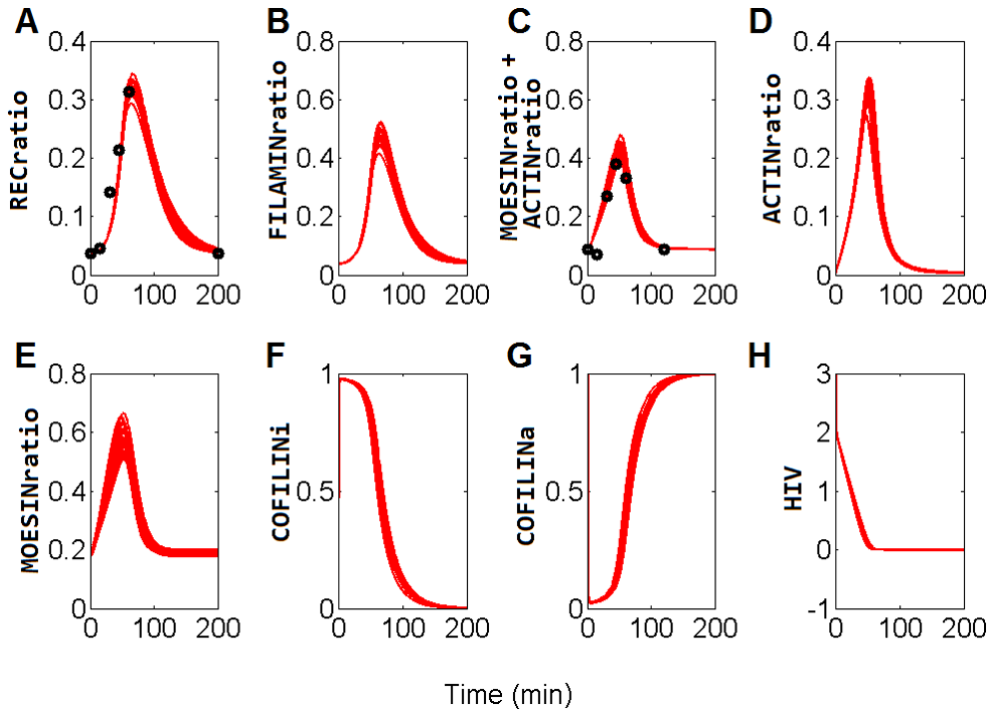
Rate constant values of the model processes. Rate constants from 1 to 13 correspond to the processes named from 1 to 13 in Figure 1. Mean values for the 12 selected solutions (see Material and Methods) are represented by the bars; standard deviation measures are included.

Valuable information can be obtained from the values of the processes' rate constants (K_n_). Their values tell us about the relative velocity of the processes. In Figure 2 it can be seen that the values of the constants for processes 3, 5, 8, 9, and 13 are almost negligible. This implies that the system dynamics is virtually independent of them.

Processes 3 and 5 represent the spontaneous aggregation and disaggregation, respectively, of filamin to the cap. It thus seems that the dynamics of filamin comes mostly from the induced effect of the receptor capping (process 4).

Process 8 represents the spontaneous disaggregation of actin from the cap. We must therefore conclude that the disaggregation of the cap is due only to the positive signaling from the HIV-1-induced molecules. This model prediction is supported by the observations of Yoder et al. 2008 [7], where they established the influence of cofilin as being determinant of subsequent stages of the invasion process.

Another process which would appear to be of little, if any, relevance is process 9, which describes the activation of moesin by causes other than the HIV-1 induction. Instead, it is the HIV-1-induced activation of moesin which is of foremost importance, as stated by Barrero-Villar et al. 2009 [10].

The last of the processes that would appear to bear little relevance to the system dynamics is the activation of cofilin (process 13). The conclusion to be drawn here is that HIV-1 infection is not due to the activation of cofilin, but rather to the induced inactivation of cofilin (process 12). It should be noted that this is the situation observed in the active lymphocytes. The importance of this process in resting lymphocytes will be analyzed below.

**Figure 3:**
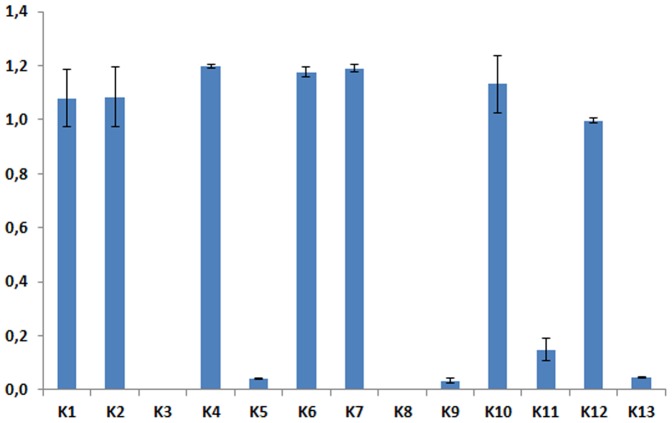
Model fitting and parameter estimation. These panels represent the 12 solutions - one for each of the predicted dynamics - which best predict the experimental ratio between total actin and total moesin as measured by Barrero-Villar et al. 2009 [10] (black solid circles). REC_ratio_: receptor ratio inside the cap; FILAMIN _ratio_: filamin-A ratio inside the cap; MOESIN _ratio_+ACTIN _ratio_: ratio of moesin within the cap over the total amount of actin and moesin, plus ratio of actin within the cap over the total amount of actin and moesin; ACTIN _ratio_: proportion of actin in the cap; MOESIN _ratio_: proportion of moesin in the cap; COFILIN_i_: inactive cofilin ratio with respect to the total amount of cofilin; COFILIN_a_: proportion of active cofilin over the total amount of cofilin; HIV: virus units per lymphocyte. The HIV variable correlates with the intensity of the signal inside the lymphocyte triggered by the virus.

The value of the rate constant of process 11 (the inactivation of moesin) deserves some attention. This constant has the larger of the low values (see Figure 2) of the rate constant. Some authors have claimed that the inactivation of moesin is necessary for the relaxation of the tension in the cap, which allows the virus to enter [10]. Our result shows that intense moesin inactivation is not a requisite for virus entry. Instead, stopping the moesin activation signaling is enough to lead to the disassembling of the actin cap (see Figure 3).

### Moesin

The actin mobilization that occurs after activation is mediated by moesin, which activates the association of actin filaments to the lymphocyte membrane at the point of the HIV-1 infection. It has been shown that this process is of foremost importance [10].

**Figure 4:**
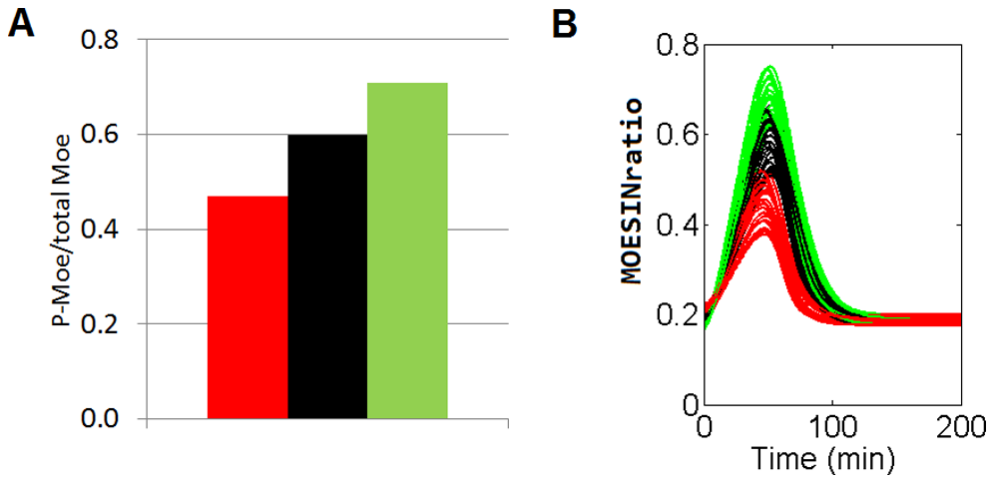
Model verification of the moesin role on the HIV-1 viral entry process. Panel A shows the total amount of functional moesin on the peak of activated moesin (at 90 minutes after infection) as determined by Barrero-Villar et al. 2009 [10]. Panel B shows the result of the MOESINratio value obtained from the model by modifying the parameter rate K_6_ (related with the total amount of moesin). The red color refers to N-Moe (a dominant negative N-terminal fragment of the protein which impedes the physiological function of the intact moesin); the black refers to the control conditions and the green to the FL-Moe (an intact form of the protein which increases the total amount of moesin inside the lymphocyte).

Our model was able to reproduce the observation made by Barrero-Villar et al. 2009 [10] regarding the role of moesin during the invasion. This work evaluates the effect of changing the total amount of functional moesin (or overexpressing a dominant negative mutant of moesin) on the peak of activated moesin. As stated above [see the Mathematical Model section], these experiments can be simulated in our model by proportionally modifying the corresponding rate parameter K_6_, which gives the total amount of functional moesin (see Supporting Information). The agreement of the model predictions regarding the moesin ratio value (Figure 4B) with the experimental maximum of the peak of activated moesin [Figure 4A] supports the reliability of our model as an integrated representation of the role of moesin in the process. We are thus provided with a suitable framework to assess the relative importance of the components of the system under different conditions.

### Gelsolin

Gelsolin, an actin-severing protein related to actin cytoskeleton reorganization, plays a role in the cortical actin reorganization during HIV-1 invasion of lymphocytes.

Although gelsolin is not explicitly represented in the model, it is possible to use the model to predict how changes in gelsolin activity will affect the dynamics of the system. This can be achieved by translating the modified values of gelsolin into the kinetic rate parameter values and observing the predicted system behavior.

To do so, two types of changes should be made simultaneously in the model. First, we should increase the amount of gelsolin. It has been proposed that gelsolin has an actin-severing activity [29], and so this increase can be mimicked in our model by increasing the kinetic rate parameter of the actin disaggregation process 7 (K_7_; see Figure 1). As an alternative to this proposed role of gelsolin, we also explored another mechanism proposed by García-Expósito et al. [14], which attributes to gelsolin a positive influence on the aggregation rate of actin to the cap. This was represented by an increase in K_6_.

At the same time, based on the observations by García-Expósito et al. [14], where it is shown that the over-expression of gelsolin decreases the total actin expression by 30%, we changed the total actin activity in the model by reducing by 30% the rate constants of processes 4 and 10, which are the processes activated by actin (see the Mathematical Model section).

**Figure 5:**
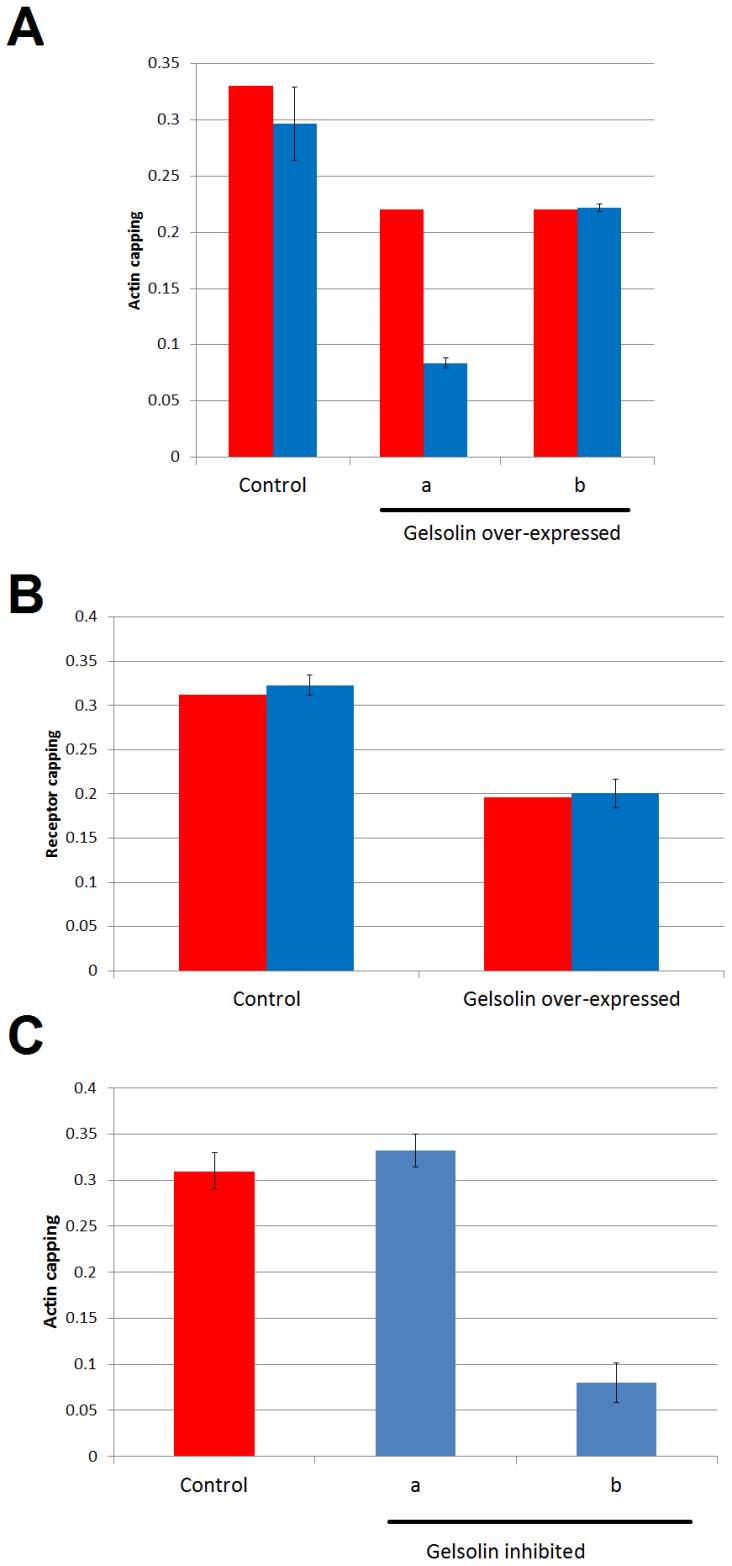
Comparison of the model predictions for two alternative roles of gelsolin in the cap formation. Red bars represent the experimental measurements of actin capping in a control situation or after over-expressing gelsolin. Blue bars represent the model prediction with the standard deviation of all solutions selected (see Material and Methods). **A.** A set of scenarios is evaluated in the model assuming different actin capping influences of gelsolin. In “a” K_7_ was increased by 50%, assuming that gelsolin has a negative effect on actin capping. In “b”, K_6_ was increased to mimic a gelsolin activation on the actin capping by increasing the actin remodeling dynamics. **B.** Model verification of scenario “b” shown in panel A. The measured and the predicted maximum peak of the capping of receptors on the gelsolin over-expressed cell lines are shown. **C.** In “a” K_6_, the parameter that models gelsolin stimulation of actin capping, takes the value of 0.75 times the value in Control; in “b” this figure is 0.5 times.

In Figure 5, the red bars show the actin capping measurements [14], while the blue bars correspond to the model predictions. Figure 5A shows the results obtained in K_7_, K_6_, K_4_ and K_10_ following the changes described above.

The “control” condition shows that the model prediction is well within the observed range of values. In scenario “a” the values of K_4_ and K_10_ have been lowered by 30% and at the same time K_7_ has been raised by 50%. In scenario “b” K_4_ and K_10_ have been lowered by 30% as before, but in this case, instead of K_7_, the other simultaneous change was in K_6_, which was increased by 27%. What can easily be observed is that there is a poor correlation between the experimental data and the data predicted by the model in scenario “a”, but that both sets of results match in scenario “b”. From these observations it can be concluded that our model supports the proposed role of gelsolin as an activator of the actin capping [14], [29]. Accordingly, it also supports the actin-severing activity of gelsolin as instrumental in facilitating the aggregation of actin by producing actin filaments of optimum sizes and the appropriate amount of these filaments to be co-localized at virus-cell contact and entry regions.

In the same vein, other evidence provided in García-Expósito et al [14] offers additional support for the model's insights into the proposed role for gelsolin. It measures the maximum peak of the capping of receptors on the gelsolin over-expressed cell lines. When the experimental data (Figure 5B) are compared with the model predictions as described above (scenario “b” of Figure 5A), a good correlation between the experimental and model results can be observed. This model verification lends additional support to the proposed effect of gelsolin on the actin capping. As a whole, we can conclude from our model that gelsolin has two direct effects on actin: one by decreasing the total amount of actin in the lymphocyte and another through promoting the aggregation of actin in the cap.

In the same work [14], it is reported that the inhibition of gelsolin negatively affects the efficiency of the virus-lymphocyte contact, and consequently impedes viral invasion. At the same time, it has been established that the amount of actin present in the cap correlates positively with infectivity [2]. In the following, we will use the actin in the cap as an indicator of the infectivity of the virus to evaluate the effect of inhibiting gelsolin in the model. As in the previous analysis, we will translate a decrease in gelsolin onto the parameters of the model in order to predict the impact of these changes on the actin capping.

Based in our previous conclusion, we can assume that a decrease in gelsolin activity will increase total actin expression (parameters K_4_ and K_10_) and reduce the rate of aggregation of actin filaments to the cap (process 6; Figure 1). In García-Expósito et al. 2013 [14], it is shown that specific knockdown of endogenous gelsolin (represented in our model as an inhibition of gelsolin function) increases the total actin expression by about 30%. Accordingly, we increased parameters K_4_ and K_10_ by 30%. Figure 5C shows the actin capping prediction after these changes. The “control” bar is the predicted actin capping level before parameter changes. In scenario “a” we see the actin capping prediction when K_4_ and K_10_ are raised by 30% and K_6_ is lowered by 25%, while in scenario “b” K_4_ and K_10_ remain the same as in “a” but K_6_ is lowered by 50%. It can be interpreted that when the effect of gelsolin inhibition on the velocity of aggregation of actin (process 6) is above 25%, the infectivity is lower than in the physiological reference conditions, a finding that correlates well with observations [14].

### Filamin-A

Filamin-A is an actin-crosslinking protein that binds to the CD4 and CXCR4 receptors after being induced by the signaling triggered by the association of the virus [15]. Interesting for the objectives of this study is the observation that the down-regulation of filamin impairs the infectivity of the virus (Jiménez-Baranda et al. 2007 [15]). Our model provides us with a tool to explore and explain this observation; for this purpose we will use, as before, the actin capping as an indicator of the infectivity. It is straightforward to simulate a decrease of the total amount of filamin-A (FILAMIN_c_) in our model. This can be done through a simultaneous decrease in the rate constants of processes 1 and 6 (K_1_ and K_6_ respectively), which are regulated by FILAMIN_c_ (see Figure 1).

**Figure 6:**
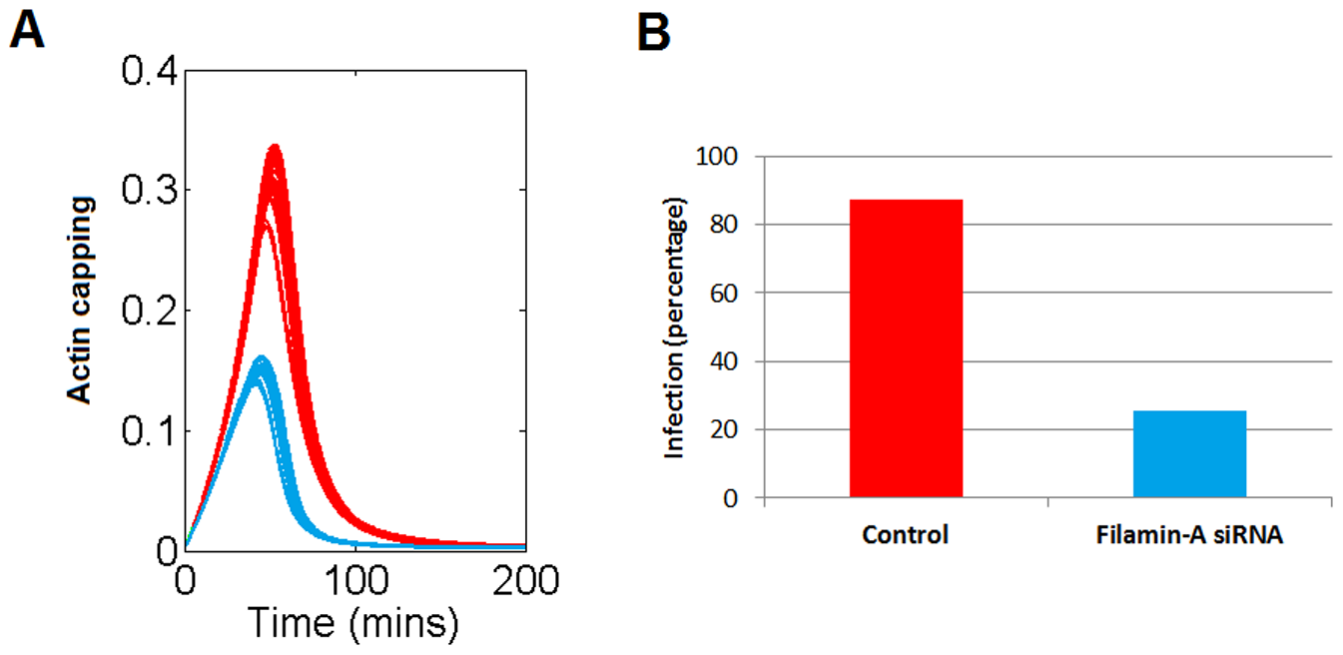
Model evaluation of the role of filamin on virus infectivity. Red curves and bars refer to the control situation, while the blue ones represent the predicted response after a decrease in the total amount of filamin-A. **A.** Virus-induced actin aggregation time course. **B.** Virus infectivity in control and filamin down-regulated conditions according to Jiménez-Baranda et al. 2007 [15].

Figure 6 shows the model prediction after simultaneous reduction in the values of K_1_ and K_6_. Figure 6A shows a decrease in the actin capping that correlates fairly well with the experimental observations (Figure 6B).

These facts constitute a mechanistic explanation, through the regulatory interactions measured by K_1_ and K_6_, of the observed reduction in the infectivity. This observation points to these regulatory interactions as potential therapeutic targets.

### Cofilin

Cofilin is another of the key players in the HIV-1 infection process. This protein initially appears in its active form (COFILIN_a_). COFILIN_a_ stimulates the disaggregation of the actin from the cap through process 7 [see Figure 1] [7], [16], thus contributing to the clearance of the cortical actin cap [7], [16]. It is only after the pore formation that the inactive [phosphorylated] form is activated (dephosphorylated) (COFILIN_i_) [7]. The inactivation of cofilin by the LIMK1 signaling pathway is represented in our model by a signal coming from HIV (process 12; Figure 1), but since this interaction has a limited temporal span, cofilin will be back in its active form at the later stages of the invasion (see panel G in Figure 3).

The model prediction on the behavior of the cofilin during the virus invasion can give us some insight into the role of this protein in the pore formation and the enhancement of HIV-1 infectivity.

Vorster et al. 2011 [22] have studied the LIMK1 signaling pathway leading to an early inactivation of cofilin, which promotes actin polymerization (see Figure 1). In the Figure 3G is represented the model predicted dynamics of the active cofilin. It can be seen the early inactivation of cofilin that rapidly falls from 1 to close to zero; behavior that tightly correlates with the sudden increase of the inactive of cofilin (Figure 3F).

**Figure 7:**
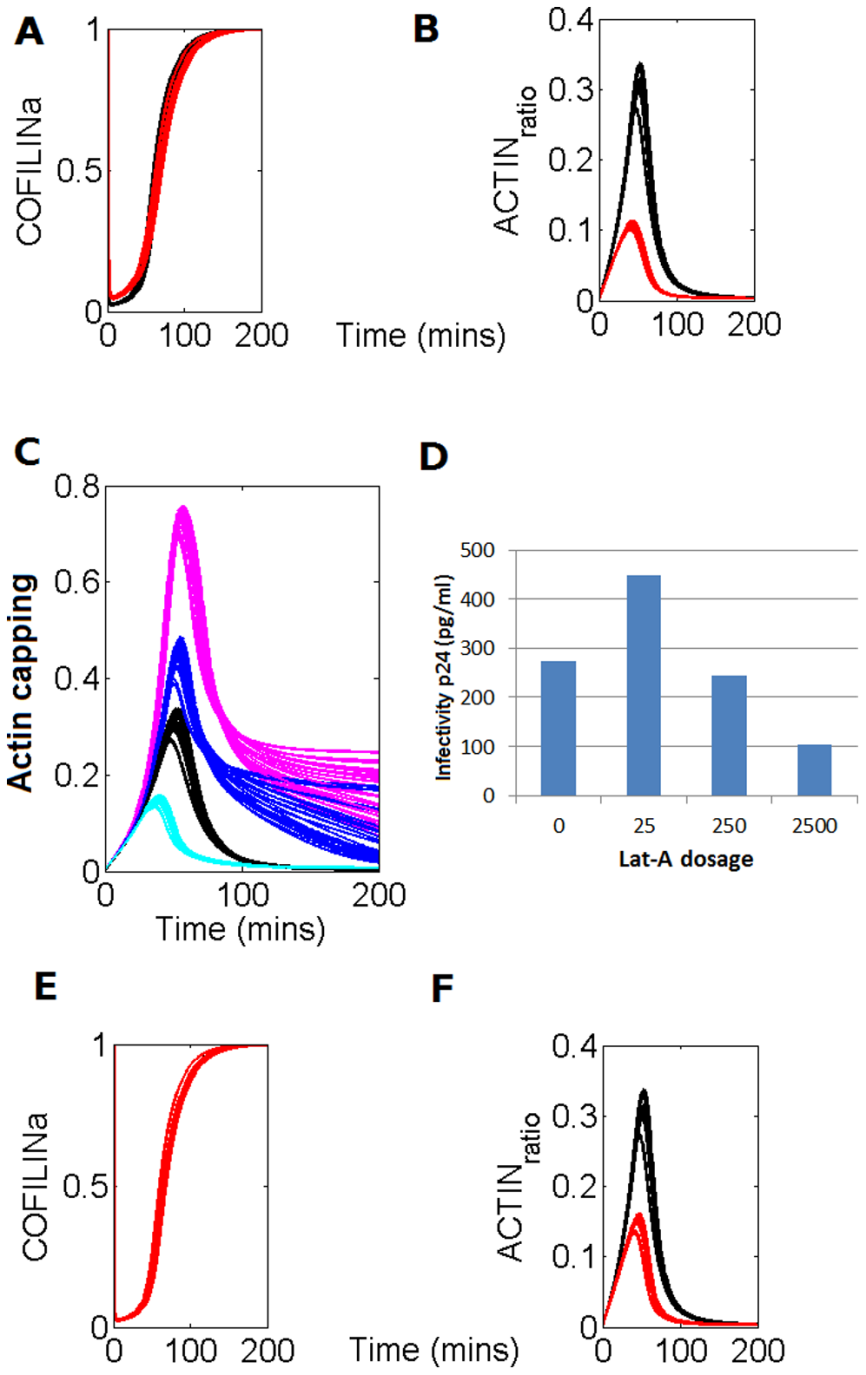
Model prediction and experimental verification of the LIMK1 signaling pathway knockdown and the actin polymerization inhibitor Lat-A on the virus infectivity. **A.** Black line displays the original solution showed in Figure 1 while the red line represents the model prediction of the COFILINa variable after inhibition of the LIMK signaling pathway by a 50%. **B.** Black line displays the original solution showed in Figure 1; red line represents the model prediction of the ACTIN variable after inhibition of the LIMK signaling pathway by a 50%. **C.** The black lines (control condition where cofilin is active before infection) show the model's predicted dynamics of the actin capping. Pink lines show the solutions obtained when the initial state of cofilin, just before infection, was inactive. Dark blue lines represent the predicted dynamics of the actin capping after the activation of virus signaling on the cofilin. Light blue lines represent an increase of the intensity of the activation signaling of cofilin by the virus **D.** Experimental measurements of infectivity of the virus in increased initial concentrations of the actin-severing factor Lat-A (Yoder et al. 2008, [7]). **E.** Black line displays the original solution showed in Figure 1 while the red line represents the model prediction of the COFILINa variable after inhibition of the WAVE2 signaling pathway by a 50%. **F.** Black line displays the original solution showed in Figure 1; red line represents the model prediction of the ACTIN variable after inhibition of the WAVE2 signaling pathway by a 50%.

Another work showed that knockdown of this LIMK1 signaling pathway decreased actin cap [23]. Again our model was able to reproduce this effect. Figure 7A and 7B displays the dynamics of the active cofilin and actin, respectively, before (black line) and after (red line) a 50% decrease in the strength of the LIMK1 signaling pathway. The model predicts a very slight decrease in the inactivation of cofilin, which, however, is enough to cause a decrease the actin cap to a third of the previous value. This prediction correlates very well with the results of Xu et al. 2012 [23] since it reproduces not only the observed fall in the peak of actin cap but also the observation of the almost negligible change in the ratio of activation of cofilin.

Yoder et al. 2008 [7], working with resting infected lymphocytes, have shown that there is a virus signaling triggered by the co-receptor that activates cofilin (process 13 in Figure 1). The same authors claim that this interaction is not present in active infected lymphocytes. In order to explain these observations, the same authors have proposed that the resting lymphocyte has a far more static cortical actin shell than the active lymphocyte [7]. Accordingly, this would be the cause of the impairment of the virus infectivity, since this cortical rigidity would impede the entry of the virus in the later stages of the invasion. In order to test this hypothesis in our model, which was built using information from experiments carried out with active infected lymphocytes [10], we have simulated a scenario in which the cortical actin situation mimics that of the resting lymphocyte. In the current model of the activated lymphocyte, it is assumed that all cofilin remains active, which also implies a very low rate of process 13. Thus, in order to test the hypothesis of Yoder et al. 2008 [7] in our model, we have to change the initial state of cofilin from active to inactive and, at the same time, to introduce a process allowing for the inactivation of cofilin in the absence of the virus. After making these changes (see Introducing an inactivation of cofilin process in Text S1), we set the rate of the new process to be 2% of the initial rate of activation of cofilin (process 13) in the activated lymphocytes in response to the virus signal. This value yields an initial inactivated cofilin of about 55% of the total actin.

Figure 7 shows the results of this exploration. In Figure 7C we see dynamics predicted by the model of the actin capping in different initial activation states of cofilin. It is observed that in conditions where cofilin remains inactive, thereby simulating a situation closer to that of a resting lymphocyte (pink curves in Figure 7C), the maximum peak of the actin present in the cap is higher than in the activated lymphocytes (black curves in Figure 7C). Also, the trend of the decay of this peak is slower when the cofilin is initially inactive as compared with the activated lymphocytes. There is additional evidence [17] that indicates that the virus invasion is less effective in resting lymphocytes than in active ones. Altogether these observations allow us to conclude that in spite of the higher peak of actin in the cap, it is the slower decay in the later stages of the invasion that serves as the restriction factor for the entry of the virus. This constitutes an “*a posteriori*”, pragmatic experimental verification of the model.

Based on the above, we made additional explorations of the effects of the co-receptor signaling on the activation of cofilin. We included in the model the activation signal on the cofilin in the case of resting lymphocytes and subsequently evaluated the effects on the dynamics of the cap of increasing values of the rate constant K_13_ (rate constant of the activation of cofilin; see Figure 1). It was observed (Figure 7C) that the delay in the actin capping decay becomes less pronounced, thus making the virus invasion more effective (dark blue lines in Figure 7C).

This observation supports the hypothesis presented by different authors [7], [22] about the role of co-receptor signaling and serves to clarify and quantify the role and importance of this signal. Also these model results correlate well with the above commented observations about the different actin dynamics and cortical F-actin amounts between non-cycling resting and cycling cell lines [8], [14], [30], [31]. In this concern, chemokine-induced actin cytoskeleton reorganization has been associated to the establishment of HIV-1 latency in infected resting CD4+ T cells [32]. An observation reinforced by the fact that inhibition of chemokine receptor-associated ability to promote intracellular signals diminishes HIV-1 infection of resting CD4+ T cells [7].

The predicted effect of actin polymerization and/or the co-receptor signals on HIV-1 infection could be verified experimentally using related published data. For example, analyzing the effect of the actin-severing activity factor latrunculin A (Lat-A) on the virus infectivity. These results, which are shown in Figure 7D (taken from Yoder et al. 2008, [7]), indicate that, in conditions of increasing dosages of the Lat-A factor, the infectivity of the virus increases after a later decay. This trend is in agreement with that predicted by our model (Figure 7C), since the increasing signal implemented in the model correlates with the increasing dosages of Lat-A: the initial increase in infectivity after a slight increase of the signal strength corresponds to a lesser delay of the actin cap. However, when the signaling intensity increases further, the model predicts a steeper decay of the actin in the cap (see light blue curves in Figure 7C), which negatively affects the invasion process and thus makes the virus infection less effective.

Considering the role of actin cytoskeleton, later after viral fusion and entry, it has been recently described that HIV-1 anchoring to CD4/CXCR4 or/CCR5 promotes transient actin polymerization in a WAVE2-Arp2/3-dependent manner, thereby favoring intracellular viral migration to the nucleus and therefore HIV-1 infection [33]–[35]. Hence, RNA interference of endogenous Arp2/3 perturbs actin nucleation and filament branching thereby diminishing viral intracellular trafficking to the nucleus and HIV-1 infection [21], [35]. These events related to Arp2/3-mediated actin dynamics on HIV-1 infection occurs later after viral entry, and merit to be analyzed in a different piece of work that could be of relevance to engage with a recently reported work that highlights the importance of the intracellular traveling of intact viral capsides for HIV-1 infection and immune escape [36]. However, it is possible with the current modelling approach, to simulate the effect on the HIV infection of the inhibition of the WAVE2 signaling. As previously indicated process 12 represents the LIMK signaling that inactivates cofilin. In fact, the WAVE2 signal bifurcates from the LIMK one, although the WAVE2 signaling activates Arp2/3 instead [35]. Process 10 is activated from the same inputs as process 12 but can also be independently activated without inactivating cofilin. On the other hand, this same process 10 activates moesin and thus, induces the aggregation of actin filaments. Since this is a similar effect that the caused by Arp2/3 we could assume that the WAVE2 signaling is represented by this process. Figure 7F shows the model prediction of the effect of reducing the WAVE2 signaling by a 50%. It can be seen that this inhibition causes a reduction of the actin aggregation, an indicator in our model of the HIV infectivity. Furthermore, Figure 7A also shows that the inactive cofilin is not altered by the WAVE2 inhibition. This result constitutes a further validation of the model reliability. We are aware that these are preliminary results which are based on assumed suppositions. Future modeling exercises on this field through the use of new data not currently available will provide further insight of the role and importance of the WAVE2 signaling.

**Figure 8:**
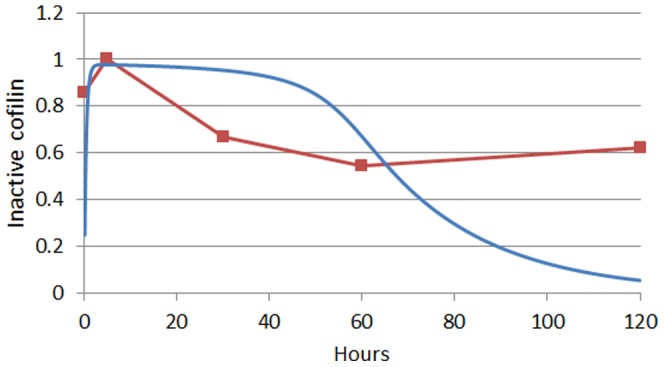
Model verification of the cofilin activity decay. Red dots and line represent the dynamics of the active cofilin during the invasion of HIV-1 as determined by Yoder et al. 2008 [7]. These observations are compared with the predicted dynamics (blue line) of the same model variable (Cof_a_).

Finally, we compared the model predictions regarding the dynamics of the inactive cofilin with the experimental observations provided by Yoder et al. 2008 [7]. In Figure 8 it can be seen that there is a decay of this protein as the invasion progresses. Here, the model prediction is close to the experimental data only in the very first moments, after which it deviates significantly. Soon after the first 10 hours the predicted cofilin is above the experimental measurements. But it turns out that, in the light of the model hypothesis, these discrepancies help us gain a better comprehension of the role of the cellular factors that are operating in the invasion process. In our simplified model, it was assumed that cofilin is the only actin-severing factor which is regulated by HIV. However, it has been proposed that another actin-severing factor such as gelsolin might play a significant role in this process [14]. It is thus suggested that the observed discrepancies could be attributed to the role that these, somewhat neglected, actin-severing HIV-1 regulated factors play during invasion.

Our results and model integrate, and also appear to predict some reported evidences that indicate that the virus invasion is less effective in resting lymphocytes than in active ones [7], [8], [17]. Moreover and considering resting lymphocytes, memory CD4+ T cells appear to be more susceptible to be infected by HIV-1 compared to naïve cells [19], [37]–[41]. Resting CD4+ T cells represent a major reservoir of HIV-1 [39], [40], being responsible for viremia when antiretroviral therapy is stopped [40]. All these data could be explained in terms of actin dynamics and cortical F-actin amount, which is different between non-cycling resting and active primary cells or cycling cell lines, with a less or a highly dynamic actin cytoskeleton, respectively [14], [30], [31]. Therefore, HIV-1-triggered actin signaling seems critical for the infection of primary CD4+ T cells.

Altogether these data and our results prompted us to propose that the our model integrates and quantifies the processes and signaling events involved in early HIV-1 infection of T cells, and supports the role and importance of HIV-1 to overcome the cortical actin barrier for efficiently fuse and infect target cells. The model also predicts the observed different susceptibility of CD4+ T cells to be infected by HIV-1 in terms of their actin cytoskeleton dynamics and the amount of cortical actin reorganized.

## Materials and Methods

### Model Design

We used the findings and observations on the processes involved in early viral entry referred to above to construct a mathematical model of these processes. This representation integrates most of the available information on the components, pathways and regulatory interactions involved in the actin rearrangements and movements during lymphocyte invasion by HIV-1. Figure 1 shows, in a schematic form, the selection of variables, processes and signaling features used.

Regarding the variables, we distinguish between those components that are integrated within the cap (denoted with subscript c) and those that are outside the cap (subscript nc). The data for the HIV variable (from now HIV without the number 1 refers to the variable of the model) have been taken from cultures of lymphocytes [10]; accordingly, this variable is expressed in units of multiplicity of infection (MOI). It should be noted that the HIV is measured as the decay of the effective virus density; thus, this variable represents the intensity of the virus signaling in a lymphocyte culture. Accordingly, the model represents the invasion at population level. Another relevant observation refers to cofilin. This protein, present in either its active or inactive form (COFILIN_a_ and COFILIN_i_, respectively), is measured in our model as the ratio of each form with respect to the total amount of cofilin present (COFILIN_a_ and COFILIN_i_).

In Figure 1, the wide arrows (numbered from 1 to 13) represent processes, while the black dashed arrows represent the referenced interactions, all of which are positive, among variables and processes.

The aggregation and disaggregation of the different forms of receptors and co-receptors (REC_nc_ and REC_c_) during the HIV-1 junction is represented by processes 1 and 2, respectively. Aggregation process 1 is activated by the cap-aggregated filamin-A (FILAMIN_c_) [15]. Processes 3 and 4 represent two different mobilization mechanisms of filamin-A to the cap. The former represents the non-regulated aggregation of filamin-A into the receptors assumed to be important, while the latter represents the aggregation of filamin-A influenced by the clustered receptors [15]. It has been shown [2], [15] that the actin form (ACTIN_c_) enhances aggregation process 4, through the interaction of filamin-A with the actin filaments. Process 5 represents the disaggregation of filamin-A from the cap.

The actin aggregation in the cap (process 6) is stimulated by filamin-A (FILAMIN_c_) [15] and moesin [10]. We assume that the inverse process, the disaggregation of the actin from the cap, can occur either spontaneously (process 8) or be promoted by active cofilin (cofilin_a_) through process 7 [7]. The spontaneous processes of association/disassociation of moesin with actin [10] are represented by processes 9 and 11. The activation of moesin by HIV-1 allows the actin to reorganize into the cell pole where the virus-cell contacts occur. This is where moesin anchors actin to the plasma membrane (this process is referred to as aggregation of moesin). In doing so, it facilitates the reorganization of CD4/CXCR4 or CCR5 receptors and the generation of the membrane tension needed for the virus-cell contacts and the fusion pore formation. During the formation of the fusion pore, moesin is inactivated, which relaxes the interaction of actin with the membrane and allows for the entry of the viral capsid. Process 10 represents the induced activation of moesin (MOESIN_nc_) with actin to become MOESIN_c_. This process is subject to two influences. One is the well-known effect of the virus on the activation process of the moesin to the cap [10]. This is represented through the interaction originating from the variable COFILIN_i_, which in turn is activated by the HIV signaling. Although this interaction has not been reported at the intracellular level, it has been observed [7], [10] that the accumulation of inactive cofilin in a culture occurs earlier than the accumulation of activated moesin. In addition, through this interaction the model is able to represent the observed time delay from the inactivation of cofilin to the activation of moesin.

On the other hand, it has been observed that, as the aggregated actin accumulates, its interaction with MOESIN_c_ intensifies, thus exerting a positive influence on the moesin association [2], [10].

Process 12 has a more complex meaning. It serves to represent the changes in the virus signaling (measured as a decay of the HIV) as well as the inactivation of COFILINa by the LIMK signaling pathway. We have chosen this form of representation since we want to illustrate the transmission of the signaling from the HIV-1 to the lymphocyte, represented as the inactivation of cofilin. Finally, process 13 represents the spontaneous transformation of the inactive cofilin (COFILIN_i_) into its active form (COFILIN_a_) [7].

### The Mathematical Model

The dynamics of the invasion process of lymphocytes by HIV-1 was represented as a set of General Mass Action [42] ordinary differential equations in a mathematical model developed within the Power Law formalism [43]. This formalism allows for non-integer kinetic orders [42], [43], but all rates were assumed to be linear. This assumption gives the formulation a mass action shape. A description of how the model equation was derived can be found in the Supporting Information (see equations S1 and S2 in Text S1).

Each of the processes described above (Figure 1; 1 to 13) has a rate kinetic constant parameter; these have been named K_1_ to K_13_. Figure 3 shows the corresponding values of the selected solutions for the model equations (mean value and standard deviation).

The regulatory interactions of a given variable on a given process (the red, dashed lines in Figure 1) are implemented in the model as having a linear effect on the corresponding rate process (see Text S1); that is, any change in the variable's value will translate proportionately to the rate of the regulated process. Accordingly, the effect of a change in the total amount of a given component in the model will be simulated in the model by changing the corresponding rate constants of the processes involved.

### Experimental Data

The modeling task refers to the initial stages of the infection dynamics just before the virus capsid is introduced inside the lymphocyte. In a culture of lymphocytes this process lasts, on average, 90 minutes [10]. However, it should be noted that the entire process can be completed in just a few seconds in an isolated lymphocyte [10]; thus the rates here have to be interpreted as population rates.

The experimental data were drawn from the work done by Barrero-Villar et al. 2009 [10]. These authors used human activated CD4+/CXCR4+ T cells to be infected by HIV-1. They collected a series of temporal data of co-localization measurements of the CD4 and CXCR4 receptors, actin and moesin. Data of the CD4 and CXCR4 receptors were used to fit the REC_c_ variable of the model, while those of actin and the ERM proteins were used to fit the sum of the variables ACTIN_c_ and MOESIN_c_. In this calculation it is assumed that moesin is a representative component of the ERM proteins [9], [10] (see Figure 3).

### Parameter Estimation

As indicated above, the model parameters were determined by fitting the model to the experimental data drawn from Barrero-Villar et al. 2009 [10] (see Figure 3). Parameters were estimated using a heuristic evolutionary optimization algorithm (Modified Genetic Algorithm), run in Matlab, previously used and presented by us elsewhere [44]–[47]. Once the maximum allowed difference between data and simulation was determined (see Parameter estimation in Text S1), a set of 12 solutions that best predicted the experimental ratio between total actin and total moesin [10] were chosen (see Figure 3). The means of the parameter values for the 12 solutions are presented in Figure 3.

### Sensitivity Analysis

Sensitivities represent the quantitative response of the model to small perturbations of physiological parameters; thus, sensitivity analysis is a powerful tool that provides a valuable indication of the robustness of a given model of system mathematical representation [48], [49]. Robustness is a general property of biological systems. Since any actual biological system is permanently exposed to perturbations from the environment, well-adapted systems should be able to conserve homeostasis. Accordingly, any useful biological model has to exhibit this property. A rule of thumb is that any sensitivity value under 1 means that the response of the system to perturbations is adequately controlled and biologically acceptable.

The sensitivity analysis yielded mean values below 0.1 for the sensitivities (see Sensitivity analysis in Text S1), which means that the model is robust enough to be considered an acceptable representation of the system.

## Conclusions

The present work is, to our knowledge, the first attempt to model and quantify the complex molecular mechanisms and the dynamics of the key variables involved in early HIV-1 infection of lymphocytes. The model, based on recent findings about these mechanisms, integrates different experimental measurements into a mathematical framework that is able to compress all considered processes simultaneously. The model's reliability was tested against new sets of experimental data not used for its calibration and parameter estimation and was submitted to sensitivity analysis for assessment of its robustness.

In spite of the data limitations, the verifications carried out on some of the model's predictions allow us to answer some key questions about the infection process and the role of the interactions involved. The results of the model confirm the important role of moesin in mobilizing and concentrating actin filaments to the contact region of the virus, by linking them to the membrane in response to virus signaling. This means that stopping moesin activation signaling is sufficient for the release of the actin filaments from the cap. Moreover, the model confirms the hypothesis proposed by García-Expósito et al. 2013 [14] by which gelsolin, as an actin-severing factor, can improve the co-localization of actin during the invasion process, thus identifying gelsolin as a promising target to impede the invasion. We also confirmed the role of filamin-A in the actin capping by linking it to the receptors influenced by the receptor capping. It is also proposed that later cofilin activation by virus signaling facilitates the entry of the virus in resting lymphocytes by accelerating the decay of the aggregated actin as a restriction factor.

Finally, the works of Barrero-Villar et al. 2009 [10], García-Expósito et al. 2013 [14] and Yoder et al. 2008 [7] put forth the idea of active cortical actin being a factor in restricting the entry of the virus. Our model supports this hypothesis, and at the same time contributes to its understanding by specifying the phenomena by which the cortical actin impedes entry. The model also proposes that there could be actin-severing factors other than cofilin induced by the virus with importance in the entry mechanism, as recently proposed for the PDZ-adaptor protein syntenin-1 that seems to similarly regulate HIV-1 entry at a post-fusion stage [50].

## Supporting Information

Figure S1 **Dynamic sensitivities respecting the initial conditions of the variables.**
(TIF)

Figure S2 **Dynamic sensitivities respecting the parameters of the model.**
(TIF)

Text S1 (DOCX)
