# Correction: Quantitative Analysis of the Processes and Signaling Events Involved in Early HIV-1 Infection of T Cells

**DOI:** 10.1371/journal.pone.0117867

**Published:** 2015-02-06

**Authors:** The PLOS ONE Staff

Some of the equations appear incorrectly in the Supporting Information file S1 Text. Please view the correct S1 Text here.

## Supporting Information

S1 Text (PDF)
