# Supplementary figures and images for: Quantitative Analysis of the Processes and Signaling Events Involved in Early HIV-1 Infection of T Cells
Source: PLoS One. 2014 Aug 8;9(8):e103845. doi: 10.1371/journal.pone.0103845 (PMC4126662; doi:10.1371/journal.pone.0103845)

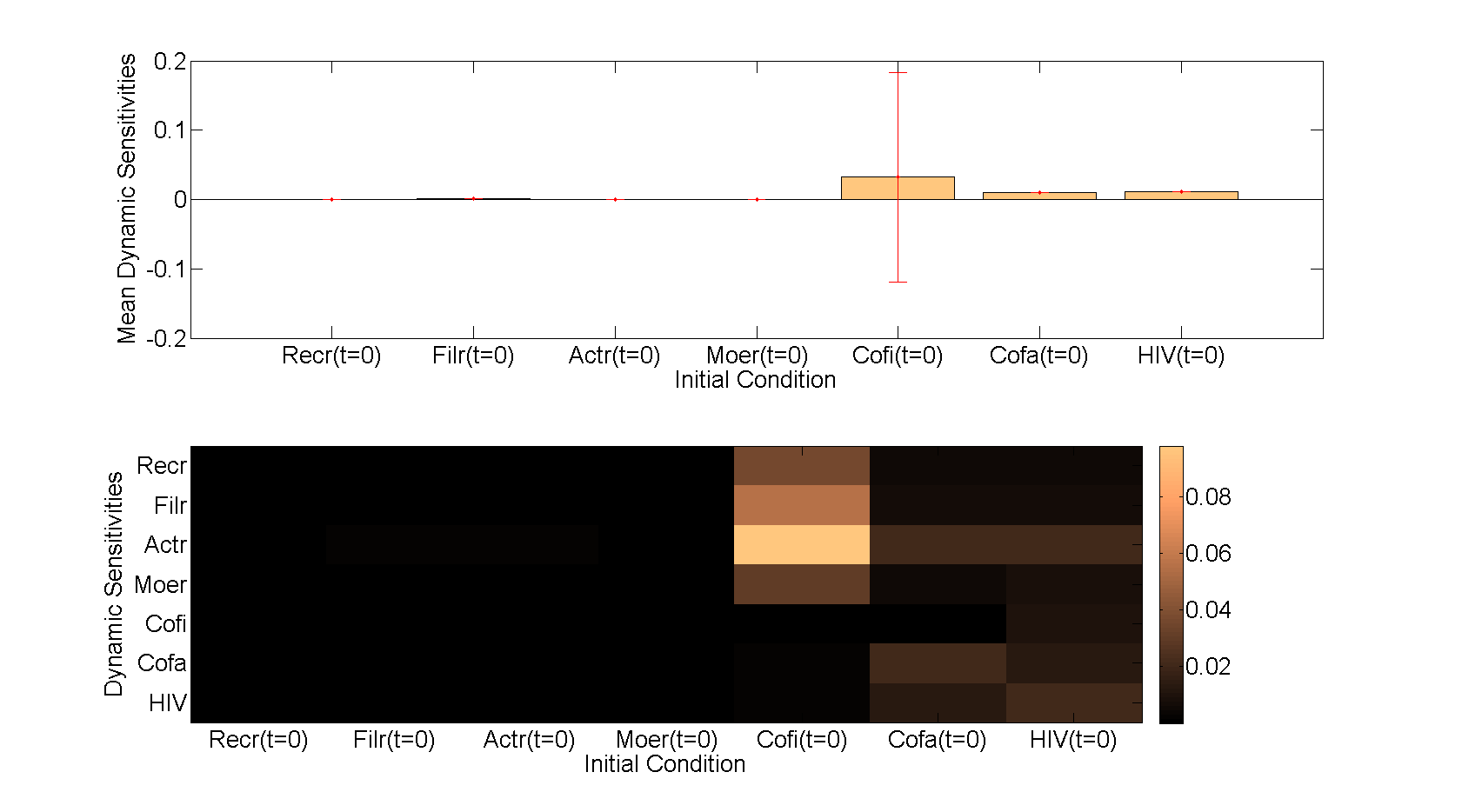

Supplement: Figure S1 — Dynamic sensitivities respecting the initial conditions of the variables. (TIF) [file pone.0103845.s001.tif]

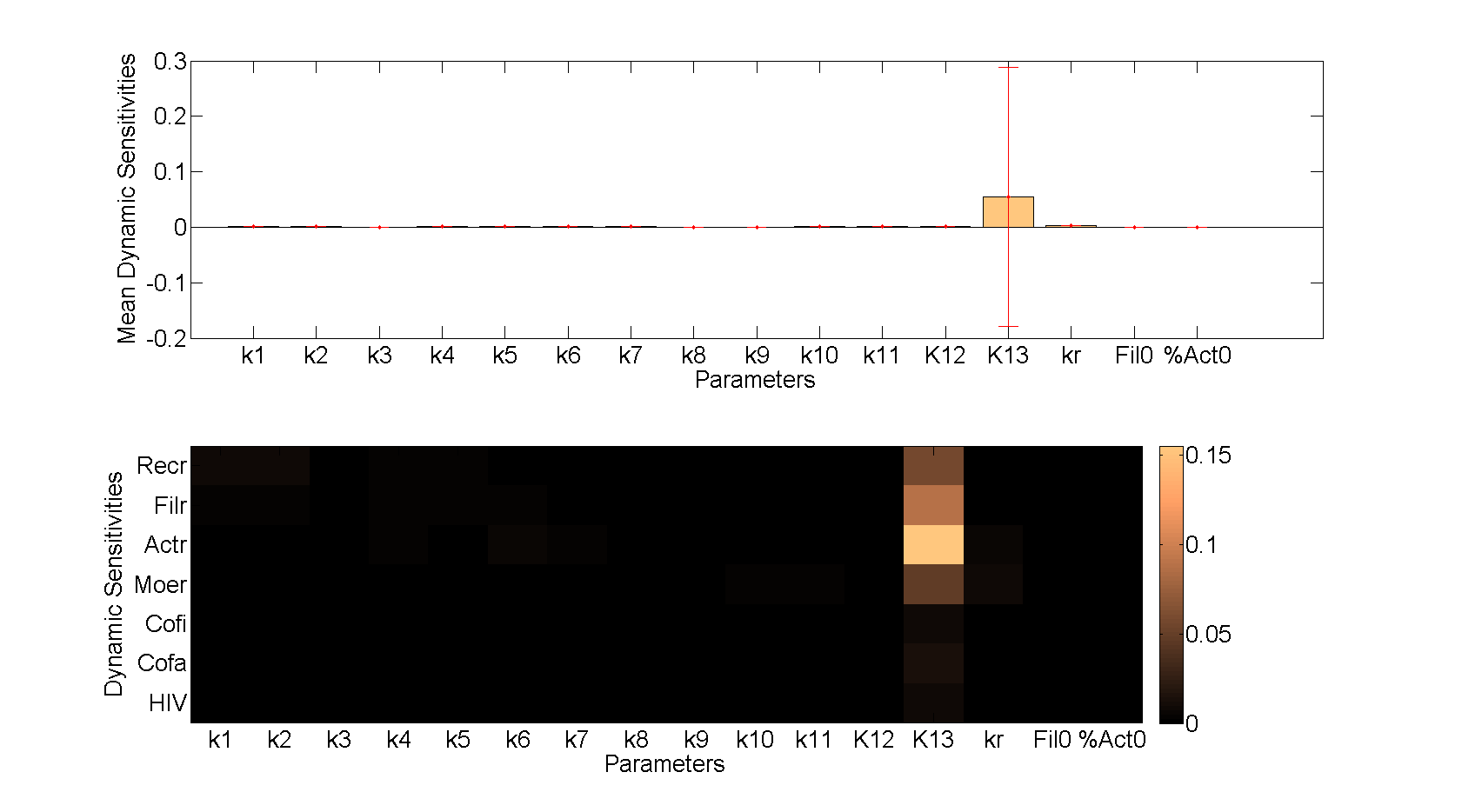

Supplement: Figure S2 — Dynamic sensitivities respecting the parameters of the model. (TIF) [file pone.0103845.s002.tif]
