# Supplementary material for: Correction: Quantitative Analysis of the Processes and Signaling Events Involved in Early HIV-1 Infection of T Cells
Source: PLoS One. 2015 Feb 6;10(2):e0117867. doi: 10.1371/journal.pone.0117867 (PMC4319938; doi:10.1371/journal.pone.0117867)
Supplement: S1 Text — (PDF) [file pone.0117867.s001.pdf]

Model equations

Equations S1 and S2 shows the mathematical model (see also equations S4, in the Model equation derivation section).

Rec represents the component receptors (CD4 and CXCR4 or CCR5) while Fil, Act and Moe stand for filamin-A, F-actin and moesin, respectively.

Since the experimental data (Barrero-Villar et al. 2009; 4) used for the parameter estimation (see the Parameter estimation section) are given as amount of each component present in the cap over the total amount of the component, the model variables are expressed also in relative values. In these cases this is denoted by the subscript *r*. The additional subscript AM in the case of Act and Moe indicates that these variables are the ratio of each component in the cap over the total amount of actin and moesin. Cof_a and Cof_i represents active and inactive cofilin, repectively. Subscript *r* means here that its value is the ratio of active or inactive cofilin over to the total amount of cofilin.

The Cofilin inactivation rate depends on Cof_{ar} and is activated by the virus signaling ($K_{12} \cdot HIV \cdot Cof_{ar}$). It is thus assumed that, during the invasion, the activation of cofilin only depends on the Cof_{ir} ($K_{13} \cdot Cof_{ir}$).

HIV represents the signaling intensity of the virus on the lymphocyte. It is measured as the proportion of lymphocytes that triggers the signal in response of virus. The model assumes that the HIV signaling inactivates cofilin and turn the signal off.

Process 12 in Figure 1 ($K_{12} \cdot HIV \cdot Cof_{ar}$) represents both, the inactivation of cofilin and the decrease in the intensity of HIV.

$$
\begin{aligned}
\frac{dRec_r}{dt} &= K_1' \cdot Fil_r - K_1' \cdot Fil_r \cdot Rec_r - K_2 \cdot Rec_r \\
\frac{dFil_r}{dt} &= K_4' \cdot Rec_r \cdot Act_{rAM} - K_4' \cdot Rec_r \cdot Act_{rAM} \cdot Fil_r + K_3 - K_3 \cdot Fil_r - K_5 \cdot Fil_r \\
\frac{dAct_{rAM}}{dt} &= (1 - K_r) \cdot K_6' \cdot Fil_r \cdot Moe_{rAM} - K_6' \cdot Fil_r \cdot Moe_{rAM} \cdot Act_{rAM} - K_7' \cdot Cof_{ar} \cdot Act_{rAM} - K_8 \cdot Act_{rAM} \\
\frac{dMoe_{rAM}}{dt} &= K_r \cdot K_9 - K_9 \cdot Moe_{rAM} + K_r \cdot K_{10}' \cdot Cof_{ir} \cdot Act_{rAM} - K_{10}' \cdot Cof_{ir} \cdot Act_{rAM} \cdot Moe_{rAM} - K_{11} \cdot Moe_{rAM} \\
\frac{dCof_{ar}}{dt} &= K_{13} \cdot Cof_{ir} - K_{12} \cdot HIV \cdot Cof_{ar} \\
\frac{dHIV}{dt} &= -K_{12} \cdot HIV \cdot Cof_{ar}
\end{aligned}
\qquad \text{Equations S1}
$$

$$X_{nc}=X_t-X_c$$
$$\frac{dX_r}{dt}=\frac{1}{X_t}\cdot\frac{dX_c}{dt}$$
$$\frac{dAct_{rAM}}{dt}=\frac{Act_t}{Act_t+Mor_t}\cdot\frac{dAct_r}{dt}$$
$$\frac{dMoe_{rAM}}{dt}=\frac{Moe_t}{Act_t+Mor_t}\cdot\frac{dMoe_r}{dt}$$
$$K_r=\frac{Moe_t}{Moe_t+Act_t}$$
$$\frac{dCof_{ir}}{dt}=\frac{-dCof_{ar}}{dt}$$
$$K_1'=K_1\cdot Fil_t$$
$$K_4'=K_4\cdot\frac{1}{1-K_r}\cdot Rec_t\cdot Act_t$$
$$K_6'=K_6\cdot\frac{1}{K_r}\cdot Fil_t\cdot Moe_t$$
$$K_7'=K_7\cdot Cof_t$$
$$K_{10}'=K_{10}\cdot\frac{1}{1-K_r}\cdot Cof_t\cdot Act_t$$

Equations S2

Parameter estimation

The model parameters (K_1 to K_{13} and K_r; K_r being the model parameter used to predict the ratio between total actin and total moesin) were with data from Barrero-Villar et al. 2009 (4). Data were presented as co-localization ratio of the components over the total amount in the cell (ERM proteins with actin, ERM proteins with CD4, REM proteins with CXCR4 and CD4 with CXCR4) during HIV invasion, measured at 0, 15, 30, 45 and 60 minutes after inoculation of the virus. Rec_r aggregates both CD4 and CXCR4 receptors. In this case the measurement of the co-localization of CD4 and CXCR4 was compared with the prediction of the variable Rec_r in the model. Moesin was used as a the representative component of the ERM proteins (Barrero-Villar et al. 2009; 4); thus co-localization data of actin and ERM proteins were used for comparison purposes with the prediction of the sum of the variables Moe_{rAM} and Act_{rAM} (see Figure 1).

Equation S3 shows the objective function used.

$$F_{obj}=\sqrt{\frac{\sum((D_{i,j}-M_{i,j})^2)}{N}}$$ Equation S3

In this equation $D_{i,j}$ represents the experimental data for time(i)=[0, 15, 30, 45, 60] (minutes), being j=1 the co-localization measure of CD4 and CXCR4 and j=2 the co-localization measure of actin and ERM proteins. $M_{i,j}$ represents the model prediction of the variable Rec for j=1 and the sum of Moe_{rAM} and Act_{rAM} for j=2. N is the number of elements of the matrix $D_{i,j}$.

In the process, the first stage was to find a model solution close enough in order to define the maximum value of the objective function. Then, one hundred solutions with objective function values lower than the previous solution were chosen. Only those solutions that best predict the experimental value of the ratio of total actin and moesin (taken from confocal imaging in Barrero-Villar et al. 2009, 4) were selected.

In order to select the solutions best predicting the ratio between total actin and total moesin we randomly sampled 10 solutions from the total and determined if the confidence

interval of K_r for the sample includes the experimental measure of the ratio of moesin over the total actin and moesin. Those solutions whose K_r confidence interval includes the experimental measure was primed and after 1000 samplings the solutions with the best scoring were selected. Only 12 solutions of the total of 100 were selected by this procedure that were used for the subsequent analysis (see Figure 2).

Sensitivity analysis

Figures S1 and S2 show the dynamic sensitivity values for the initial conditions and parameters, respectively.

Introducing an inactivation of cofilin process

To make the model able to simulate a resting lymphocyte we changed the model in order to represent that condition present of the resting lymphocyte which is relevant for the scope of the model, namely the mostly inactivated state of the cofilin (10). The model is initially in a steady situation, where cofilin is activated (see Figure 2), but when the HIV triggers the signaling, cofilin begins to decrease. To change the initial steady value of active cofilin, process 13, which denotes the rate of activation of cofilin, has to be decreased since this change will cause the initial steady value of active cofilin decrease. We set up the value of 55% of activated cofilin at the beginning; a value that can be obtained by decreasing the rate of process 13 down to 2% of the initial value.

Model derivation

Equations S4 shows the first mathematical model presentation that results from the direct translation of the mechanistic model represented in Figure 1 to the Generalized Mass Action formalism (18).

In this representation variables not belonging to the cap are represented as the difference between total minus the amount in the cap (see Equations S5).

$$\frac{dRec_c}{dt} = K_1 \cdot Fil_c \cdot Rec_{nc} - K_2 \cdot Rec_c$$

$$\frac{dFil_c}{dt} = K_4 \cdot Rec_c \cdot Act_c \cdot Fil_{nc} + K_3 \cdot Fil_{nc} - K_5 \cdot Fil_c$$

$$\frac{dAct_c}{dt} = K_6 \cdot Fil_c \cdot Moe_c \cdot Act_{nc} - K_7 \cdot Cof_a \cdot Act_c - K_8 \cdot Act_c$$

$$\frac{dMoe_c}{dt} = K_9 \cdot Moe_{nc} + K_{10} \cdot Cof_i \cdot Act_c \cdot Moe_{nc} - K_{11} \cdot Moe_c$$

$$\frac{dCof_a}{dt} = K_{13} \cdot Cof_i - K_{12} \cdot HIV \cdot Cof_a$$

$$\frac{dHIV}{dt} = -K_{12} \cdot HIV \cdot Cof_a$$

Equations S4

$$\frac{dRec_c}{dt} = K_1 \cdot Fil_c \cdot (Rec_t - Rec_c) - K_2 \cdot Rec_c$$
$$\frac{dFil_c}{dt} = K_4 \cdot Rec_c \cdot Act_c \cdot (Fil_t - Fil_c) + K_3 \cdot (Fil_t - Fil_c) - K_5 \cdot Fil_c$$
$$\frac{dAct_c}{dt} = K_6 \cdot Fil_c \cdot Moe_c \cdot (Act_t - Act_c) - K_7 \cdot Cof_a \cdot Act_c - K_8 \cdot Act_c$$
$$\frac{dMoe_c}{dt} = K_9 \cdot (Moe_t - Moe_c) + K_{10} \cdot Cof_i \cdot Act_c \cdot (Moe_t - Moe_c) - K_{11} \cdot Moe_c$$
$$\frac{dCof_a}{dt} = K_{13} \cdot Cof_i - K_{12} \cdot HIV \cdot Cof_a$$
$$\frac{dHIV}{dt} = -K_{12} \cdot HIV \cdot Cof_a$$

Equations S5

In order to represents the variables in relative values each variable is divided by the total amount of each component (Equations S6).

$$\frac{dRec_r}{dt} = K_1 \cdot \frac{Fil_t}{Fil_t} \cdot Fil_c - K_1 \cdot \frac{Fil_t}{Fil_t} \cdot Fil_c \cdot Rec_r - K_2 \cdot Rec_r$$
$$\frac{dFil_r}{dt} = K_4 \cdot \frac{Rec_t}{Rec_t} \cdot Rec_c \cdot \frac{Act_t}{Act_t} \cdot Act_c - K_4 \cdot \frac{Rec_t}{Rec_t} \cdot Rec_c \cdot \frac{Act_t}{Act_t} \cdot Act_c \cdot Fil_r + K_3 - K_3 \cdot Fil_r - K_5 \cdot Fil_r$$
$$\frac{dAct_r}{dt} = K_6 \cdot \frac{Fil_t}{Fil_t} \cdot Fil_c \cdot \frac{Moe_t}{Moe_t} \cdot Moe_c - K_6 \cdot \frac{Fil_t}{Fil_t} \cdot Fil_c \cdot \frac{Moe_t}{Moe_t} \cdot Moe_c \cdot Act_r - K_7 \cdot \frac{Cof_t}{Cof_t} \cdot Cof_a \cdot Act_r - K_8 \cdot Act_r$$
$$\frac{dMoe_r}{dt} = K_9 \cdot - K_9 \cdot Moe_r + K_{10} \cdot \frac{Cof_t}{Cof_t} \cdot Cof_i \cdot \frac{Act_t}{Act_t} \cdot Act_c - K_{10} \cdot \frac{Cof_t}{Cof_t} \cdot Cof_i \cdot \frac{Act_t}{Act_t} \cdot Act_c \cdot Moe_r - K_{11} \cdot Moe_r$$
$$\frac{dCof_{ar}}{dt} = K_{13} \cdot Cof_{ir} - K_{12} \cdot HIV \cdot Cof_{ar}$$
$$\frac{dHIV}{dt} = -K_{12} \cdot HIV \cdot Cof_{ar}$$

Equations S6

By substitution of the variables as indicated in the Model equations paragraph we obtain Equations S7.

$$\frac{dRec_r}{dt} = K_1 \cdot Fil_t \cdot Fil_r - K_1 \cdot Fil_t \cdot Fil_r \cdot Rec_r - K_2 \cdot Rec_r$$
$$\frac{dFil_r}{dt} = K_4 \cdot Rec_t \cdot Act_t \cdot Rec_r \cdot Act_r - K_4 \cdot Rec_t \cdot Act_t \cdot Rec_r \cdot Act_r \cdot Fil_r + K_3 - K_3 \cdot Fil_r - K_5 \cdot Fil_r$$
$$\frac{dAct_r}{dt} = K_6 \cdot Fil_t \cdot Moe_t \cdot Fil_r \cdot Moe_r - K_6 \cdot Fil_t \cdot Moe_t \cdot Fil_r \cdot Moe_r \cdot Act_r - K_7 \cdot Cof_t \cdot Cof_{ar} \cdot Act_r - K_8 \cdot Act_r$$
$$\frac{dMoe_r}{dt} = K_9 - K_9 \cdot Moe_r + K_{10} \cdot Cof_t \cdot Act_t \cdot Cof_{ir} \cdot Act_r - K_{10} \cdot Cof_t \cdot Act_t \cdot Cof_{ir} \cdot Act_r \cdot Moe_r - K_{11} \cdot Moe_r$$
$$\frac{dCof_{ar}}{dt} = K_{13} \cdot Cof_{ir} - K_{12} \cdot HIV \cdot Cof_{ar}$$
$$\frac{dHIV}{dt} = -K_{12} \cdot HIV \cdot Cof_{ar}$$

Equations S7

Due to the nature of the experimental data used (4) variables Moe and Act are normalized

by dividing by the summation of Moe and Act. Equation S8, S9 and S10 show the procedure followed.

$$\frac{dRec_r}{dt}=K_1\cdot Fil_t\cdot Fil_r-K_1\cdot Fil_t\cdot Fil_r\cdot Rec_r-K_2\cdot Rec_r$$
$$\frac{dFil_r}{dt}=K_4\cdot Rec_t\cdot Act_t\cdot Rec_r\cdot Act_r-K_4\cdot Rec_t\cdot Act_t\cdot Rec_r\cdot Act_r\cdot Fil_r+K_3-K_3\cdot Fil_r-K_5\cdot Fil_r$$
$$\frac{Act_t}{Act_t+Mor_t}\cdot\frac{dAct_r}{dt}=\frac{Act_t}{Act_t+Mor_t}\cdot\left(K_6\cdot Fil_t\cdot Moe_t\cdot Fil_r\cdot Moe_r-K_6\cdot Fil_t\cdot Moe_t\cdot Fil_r\cdot Moe_r\cdot Act_r-K_7\cdot Cof_t\cdot Cof_{ar}\cdot Act_r-K_8\cdot Act_r\right)$$
$$\frac{Moe_t}{Act_t+Mor_t}\cdot\frac{dMoe_r}{dt}=\frac{Moe_t}{Act_t+Mor_t}\cdot\left(K_9-K_9\cdot Moe_r+K_{10}\cdot Cof_t\cdot Act_t\cdot Cof_{ir}\cdot Act_r-K_{10}\cdot Cof_t\cdot Act_t\cdot Cof_{ir}\cdot Act_r\cdot Moe_r-K_{11}\cdot Moe_r\right)$$
$$\frac{dCof_{ar}}{dt}=K_{13}\cdot Cof_{ir}-K_{12}\cdot HIV\cdot Cof_{ar}$$
$$\frac{dHIV}{dt}=-K_{12}\cdot HIV\cdot Cof_{ar}$$

Equations S8

$$\frac{dRec_r}{dt}=K_1\cdot Fil_t\cdot Fil_r-K_1\cdot Fil_t\cdot Fil_r\cdot Rec_r-K_2\cdot Rec_r$$
$$\frac{dFil_r}{dt}=K_4\cdot Rec_t\cdot Act_t\cdot Rec_r\cdot\frac{Act_t+Mor_t}{Act_t}\cdot Act_{rAM}-K_4\cdot Rec_t\cdot Act_t\cdot Rec_r\cdot\frac{Act_t+Mor_t}{Act_t}\cdot Act_{rAM}\cdot Fil_r+K_3-K_3\cdot Fil_r-K_5\cdot Fil_r$$
$$\frac{dAct_{rAM}}{dt}=\frac{Act_t}{Act_t+Mor_t}\cdot\left(K_6\cdot Fil_t\cdot Moe_t\cdot Fil_r\cdot\frac{Moe_t+Mor_t}{Act_t}\cdot Moe_{rAM}-K_6\cdot Fil_t\cdot Moe_t\cdot Fil_r\cdot\frac{Moe_t+Mor_t}{Act_t}\cdot Moe_{rAM}\cdot\frac{Act_t+Mor_t}{Act_t}\cdot Act_{rAM}-K_7\cdot Cof_t\cdot Cof_{ar}\cdot\frac{Act_t+Mor_t}{Act_t}\cdot Act_{rAM}-K_8\cdot\frac{Act_t+Mor_t}{Act_t}\cdot Act_{rAM}\right)$$
$$\frac{dMoe_{rAM}}{dt}=\frac{Moe_t}{Act_t+Mor_t}\cdot\left(K_9-K_9\cdot\frac{Moe_t+Mor_t}{Act_t}\cdot Moe_{rAM}+K_{10}\cdot Cof_t\cdot Act_t\cdot Cof_{ir}\cdot\frac{Act_t+Mor_t}{Act_t}\cdot Act_{rAM}-K_{10}\cdot Cof_t\cdot Act_t\cdot Cof_{ir}\cdot\frac{Act_t+Mor_t}{Act_t}\cdot Act_{rAM}\cdot\frac{Moe_t+Mor_t}{Act_t}\cdot Moe_{rAM}-K_{11}\cdot\frac{Moe_t+Mor_t}{Act_t}\cdot Moe_{rAM}\right)$$
$$\frac{dCof_{ar}}{dt}=K_{13}\cdot Cof_{ir}-K_{12}\cdot HIV\cdot Cof_{ar}$$
$$\frac{dHIV}{dt}=-K_{12}\cdot HIV\cdot Cof_{ar}$$

Equations S9

$$\frac{dRec_r}{dt}=K_1\cdot Fil_t\cdot Fil_r-K_1\cdot Fil_t\cdot Fil_r\cdot Rec_r-K_2\cdot Rec_r$$
$$\frac{dFil_r}{dt}=K_4\cdot Rec_t\cdot Act_t\cdot Rec_r\cdot\frac{Act_t+Moe_t}{Act_t}\cdot Act_{rAM}-K_4\cdot Rec_t\cdot Act_t\cdot Rec_r\cdot\frac{Act_t+Moe_t}{Act_t}\cdot Act_{rAM}\cdot Fil_r+K_3-K_3\cdot Fil_r-K_5\cdot Fil_r$$
$$\frac{dAct_{rAM}}{dt}=\frac{Act_t}{Act_t+Moe_t}\cdot K_6\cdot Fil_t\cdot Moe_t\cdot Fil_r\cdot\frac{Moe_t+Act_t}{Moe_t}\cdot Moe_{rAM}-K_6\cdot Fil_t\cdot Moe_t\cdot Fil_r\cdot\frac{Moe_t+Act_t}{Moe_t}\cdot Moe_{rAM}\cdot Act_{rAM}-K_7\cdot Cof_t\cdot Cof_{ar}\cdot Act_{rAM}-K_8\cdot Act_{rAM}$$
$$\frac{dMoe_{rAM}}{dt}=\frac{Moe_t}{Act_t+Moe_t}\cdot K_9-K_9\cdot Moe_{rAM}+K_{10}\cdot Cof_t\cdot Act_t\cdot Cof_{ir}\cdot\frac{Moe_t}{Act_t+Moe_t}\cdot\frac{Act_t+Moe_t}{Act_t}\cdot Act_{rAM}-K_{10}\cdot Cof_t\cdot Act_t\cdot Cof_{ir}\cdot\frac{Act_t+Moe_t}{Act_t}\cdot Act_{rAM}\cdot Moe_{rAM}-K_{11}\cdot Moe_{rAM}$$
$$\frac{dCof_{ar}}{dt}=K_{13}\cdot Cof_{ir}-K_{12}\cdot HIV\cdot Cof_{ar}$$
$$\frac{dHIV}{dt}=-K_{12}\cdot HIV\cdot Cof_{ar}$$

Equations S10

K_r is a model constant representing the constant ratio value of total moesin over the summation of total moesin and actin.

$$\frac{dRec_r}{dt}=K_1\cdot Fil_t\cdot Fil_r-K_1\cdot Fil_t\cdot Fil_r\cdot Rec_r-K_2\cdot Rec_r$$
$$\frac{dFil_r}{dt}=K_4\cdot\frac{1}{1-K_r}\cdot Rec_t\cdot Act_t\cdot Rec_r\cdot Act_{rAM}-K_4\cdot\frac{1}{1-K_r}\cdot Rec_t\cdot Act_t\cdot Rec_r\cdot Act_{rAM}\cdot Fil_r+K_3-K_3\cdot Fil_r-K_5\cdot Fil_r$$
$$\frac{dAct_{rAM}}{dt}=\frac{(1-K_r)}{K_r}\cdot K_6\cdot Fil_t\cdot Moe_t\cdot Fil_r\cdot Moe_{rAM}-K_6\cdot\frac{1}{K_r}\cdot Fil_t\cdot Moe_t\cdot Fil_r\cdot Moe_{rAM}\cdot Act_{rAM}-K_7\cdot Cof_t\cdot Cof_{ar}\cdot Act_{rAM}-K_8\cdot Act_{rAM}$$
$$\frac{dMoe_{rAM}}{dt}=K_r\cdot K_9-K_9\cdot Moe_{rAM}+\frac{K_r}{1-K_r}\cdot K_{10}\cdot Cof_t\cdot Act_t\cdot Cof_{ir}\cdot Act_{rAM}-K_{10}\cdot\frac{1}{1-K_r}\cdot Cof_t\cdot Act_t\cdot Cof_{ir}\cdot Act_{rAM}\cdot Moe_{rAM}-K_{11}\cdot Moe_{rAM}$$
$$\frac{dCof_{ar}}{dt}=K_{13}\cdot Cof_{ir}-K_{12}\cdot HIV\cdot Cof_{ar}$$
$$\frac{dHIV}{dt}=-K_{12}\cdot HIV\cdot Cof_{ar}$$

Equations S11

The total amount of each variable, that also remains constant are grouped all together in the constant (K_n).

$$\frac{dRec_r}{dt}=K_1'\cdot Fil_r-K_1'\cdot Fil_r\cdot Rec_r-K_2\cdot Rec_r$$
$$\frac{dFil_r}{dt}=K_4'\cdot Rec_r\cdot Act_{rAM}-K_4'\cdot Rec_r\cdot Act_{rAM}\cdot Fil_r+K_3-K_3\cdot Fil_r-K_5\cdot Fil_r$$
$$\frac{dAct_{rAM}}{dt}=(1-K_r)\cdot K_6'\cdot Fil_r\cdot Moe_{rAM}-K_6'\cdot Fil_r\cdot Moe_{rAM}\cdot Act_{rAM}-K_7'\cdot Cof_{ar}\cdot Act_{rAM}-K_8\cdot Act_{rAM}$$
$$\frac{dMoe_{rAM}}{dt}=K_r\cdot K_9-K_9\cdot Moe_{rAM}+K_r\cdot K_{10}'\cdot Cof_{ir}\cdot Act_{rAM}-K_{10}'\cdot Cof_{ir}\cdot Act_{rAM}\cdot Moe_{rAM}-K_{11}\cdot Moe_{rAM}$$
$$\frac{dCof_{ar}}{dt}=K_{13}\cdot Cof_{ir}-K_{12}\cdot HIV\cdot Cof_{ar}$$
$$\frac{dHIV}{dt}=-K_{12}\cdot HIV\cdot Cof_{ar}$$

Equations S12

$$X_{nc}=X_t-X_c$$
$$\frac{dX_r}{dt}=\frac{1}{X_t}\cdot\frac{dX_c}{dt}$$
$$\frac{dAct_{rAM}}{dt}=\frac{Act_t}{Act_t+Mor_t}\cdot\frac{dAct_r}{dt}$$
$$\frac{dMoe_{rAM}}{dt}=\frac{Moe_t}{Act_t+Mor_t}\cdot\frac{dMoe_r}{dt}$$
$$K_r=\frac{Moe_t}{Moe_t+Act_t}$$
$$\frac{dCof_{ir}}{dt}=\frac{-dCof_{ar}}{dt}$$
$$K_1'=K_1\cdot Fil_t$$
$$K_4'=K_4\cdot\frac{1}{1-K_r}\cdot Rec_t\cdot Act_t$$
$$K_6'=K_6\cdot\frac{1}{K_r}\cdot Fil_t\cdot Moe_t$$
$$K_7'=K_7\cdot Cof_t$$
$$K_{10}'=K_{10}\cdot\frac{1}{1-K_r}\cdot Cof_t\cdot Act_t$$

Equations S13
